# Imported and travelling dogs as carriers of canine vector-borne pathogens in Germany

**DOI:** 10.1186/1756-3305-3-34

**Published:** 2010-04-08

**Authors:** Brigitte Menn, Susanne Lorentz, Torsten J Naucke

**Affiliations:** 1 Institute for Zoomorphology, Cytology and Parasitology, Heinrich Heine University, Universitätsstraße 1, 40225 Düsseldorf, Germany; 2 Parasitus Ex e.V., Vollbergstraße 37, 53859 Niederkassel, Germany; 3 Department of Zoology, Division of Parasitology, University of Hohenheim, 70599 Stuttgart, Germany; 4 Laboklin GmbH & Co. KG, Steubenstr. 4, 97688 Bad Kissingen, Germany

## Abstract

**Background:**

With the import of pets and pets taken abroad, arthropod-borne diseases have increased in frequency in German veterinary practices. This is reflected by 4,681 dogs that have been either travelled to or relocated from endemic areas to Germany. The case history of these dogs and the laboratory findings have been compared with samples collected from 331 dogs living in an endemic area in Portugal. The various pathogens and the seroprevalences were examined to determine the occurrence of, and thus infection risk, for vector-borne pathogens in popular travel destinations.

**Results:**

4,681 dogs were examined serological for *Leishmania infantum, Babesia canis *and *Ehrlichia canis*. Buffy coats were detected for *Hepatozoon canis *and blood samples were examined for microfilariae via the Knott's test. The samples were sent in from animal welfare organizations or private persons via veterinary clinics. Upon individual requests, dogs were additionally examined serological for *Anaplasma phagocytophilum, Borrelia burgdorferi *and *Rickettsia conorii*. Overall *B. canis *was the most prevalent pathogen detected by antibody titers (23.4%), followed by *L. infantum *(12.2%) and *E. canis *(10.1%). Microfilariae were detected in 7.7% and *H. canis *in 2.7% of the examined dogs. In 332/1862 dogs *A. phagocytophilum*, in 64/212 *B. burgdorferi *and in 20/58 *R. conorii *was detected. Of the 4,681 dogs, in total 4,226 were imported to Germany from endemic areas. Eighty seven dogs joined their owners for a vacation abroad. In comparison to the laboratory data from Germany, we examined 331 dogs from Portugal. The prevalence of antibodies/pathogens we detected was: 62.8% to *R. conorii*, 58% to *B. canis*, 30.5% to *A. phagocytophilum*, 24.8% to *E. canis*, 21.1% to *H. canis *(via PCR), 9.1% to *L. infantum *and 5.3% to microfilariae.

**Conclusions:**

The examination of 4,681 dogs living in Germany showed pathogens like *L. infantum *that are non-endemic in Germany. Furthermore, the German data are similar in terms of multiple pathogen infection to the data recorded for dogs from Portugal. Based on these findings the importation of dogs from endemic predominantly Mediterranean regions to Germany as well as travelling with dogs to these regions carries a significant risk of acquiring an infection. Thus we would conclude that pet owners seek advice of the veterinarians prior to importing a dog from an endemic area or travel to such areas. In general, it might be advisable to have a European recording system for translocation of dogs.

## Background

The zoogeographical range of pathogens of arthropod-borne diseases is restricted by the distribution areas of their vectors and hosts [1]. Dogs are competent reservoir hosts of several zoonotic pathogens and can serve as a readily available source of nutrition for many blood-feeding arthropods [2]. Increasing pet tourism and importation of animals from endemic areas present German veterinary practitioners increasingly with exotic diseases, like leishmaniosis, babesiosis, ehrlichiosis and dirofilariosis [3-7]. The frequency of dog-tourism and -import was first reported in the study of Glaser and Gothe, who analyzed 5,340 questionnaires in the years 1985 to 1995 [4]. The results revealed a steady increase of dogs taken abroad, rising from 31.1% in 1990 to 40.8% in 1994. Also in the United Kingdom an increasingly mobility of pets is conspicuous. Since February 2000 every pet entering the United Kingdom is registered in conjunction with the Pet Travel Scheme (PETS) and the released data show a steadily increase from 14,695 pets in the year 2000 up to 82,674 pets in the year 2006 [8,1]. Besides the registration of departure and entry, pets have to run through a serology and ecto- and endoparasiticidal treatment 24-48 h before re-entry to the United Kingdom [1]. This is important, because pets travelling abroad are exposed to various arthropod-borne diseases, especially in the popular destinations of the Mediterranean area and Portugal [4,7,9]. In addition to the pets joining their owners for a vacation, a large number of dogs, is imported to Germany by tourists or animal protection societies [3,4,10,11]. While born and raised in the endemic area - their country of origin - imported dogs have an increased risk of contracting a canine vector-borne disease (CVBD) [5].

National and international investigations are necessary to be able to estimate topical risks, both in endemic and in currently non-endemic regions. This information would suggest how to avoid an import of pathogens, e.g. with the help of preventive measures. The increased mobility of pets is an important matter in the extension of the zoogeographical ranges for many arthropod-borne pathogens [1]. A previously non-endemic region may become endemic tomorrow. This risk is supported by the first autochthonous cases in Germany published for infections with *H. canis *[12], *L. infantum *[13], *E. canis *[14] and *D. repens *[15,16]. These are pathogens of traditional so called travel-related diseases.

To obtain an overview of the situation of travelling, and particularly imported dogs, the results of the diagnosed 4,681 dog samples between July 2004 and December 2009 are analyzed epidemiologically- including information of origin countries and length of vacation. To compare the data from non-endemic diseases in Germany a randomly selected endemic area in Portugal was selected. Blood- samples of 331 dogs from Portugal were examined during the years 2007 and 2008 for examination of CVBD pathogens and their seroprevalences.

## Results

In the present study we included the findings from 4,681 dog blood samples collected between July 2004 and December 2009 and additional 331 samples from Portuguese dogs on the occurrence of single and multiple infections of the following CVBD's: *L. infantum, E. canis, B. canis*, microfilariae and *H. canis*. *L. infantum, E. canis *and *B. canis *were detected serological using the Immunofluorescence Antibody Test (IFAT). All samples were examined for microfilariae using the Knott's test and buffy coats were detected for gamonts of *H. canis*. The 331 Portuguese samples were additionally examined for *H. canis *via PCR. *A. phagocytophilum *and *R. conorii *were detected serological in the Portuguese and in 1862 and 58 samples of the laboratory diagnosed data. Additional 212 samples of the laboratory diagnosed data were examined serological for *B. burgdorferi*.

### Results of the 4,681 samples diagnosed from July, 2004 to December, 2009

4,226 of the 4,681 were imported dogs from various endemic regions (90.3%). Eighty-seven dogs were of German origin and accompanied their owners for vacation to endemic areas (1.8%). For 368 dogs, or 7.9% of the sample, the documentation sheet was incomplete, thus these dogs could not be allocated to either other group.

From the total of 4,226 imported dogs, 2,906 (68.8%) were born either in Portugal (n = 928) or in countries bordering the Mediterranean, especially Spain (n = 1,162), Italy (n = 367), Greece (n = 267) and Turkey (n = 106), but also in France (n = 37), Malta (n = 18), Croatia (n = 17) and Slovenia (n = 4).

A total of 1,320 (31.2%) of the 4,226 imported dogs were born in European countries beyond the Mediterranean region, mostly in Hungary (n = 1,013) and Romania (n = 279). Twenty-eight other dogs were born in Bulgaria (n = 14), Poland (n = 8), Switzerland (n = 2), Denmark (n = 1), Austria (n = 1), Holland (n = 1) and Czech Republic (n = 1).

78.2% of 87 dogs which had accompanied their owners abroad, travelled to Mediterranean countries: Spain (n = 22), Italy (n = 21), France (n = 10), Turkey (n = 8), Croatia (n = 3), Greece (n = 3) and Portugal (n = 1). Less than a quarter of the dogs (21.8%) traveled to Hungary (n = 7), Austria (n = 3), Denmark (n = 3), Switzerland (n = 2), Belgium (n = 1), Czech Republic (n = 1), Great Britain (n = 1) and Holland (n = 1).

**Figure 1:**
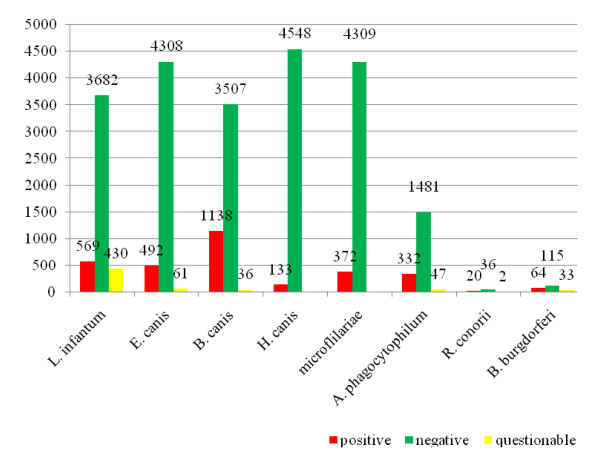
**Number of pathogens detected by IFAT, BC and Knott's test in 4,681 German dogs send in from animal welfare organizations and private persons between July 2004 and December 2009**. Numbers of positive, negative and questionable test results of a total of 4,681 dogs sent in from animal welfare organizations and private persons. Blood samples were examined by means of Knott's test for microfilariae. The samples were tested on *H. canis *with the help of the examination of the buffy coats (BC). The seroprevalences of *B. canis, E. canis *and *L. infantum *were determined by means of Immunofluorescence Antibody Test (IFAT). In 1,862 cases the seroprevalence of *A. phagocytophilum*, in 212 cases of *B. burgdorferi *and in 58 cases of *R. conorii *were examined.

The prevalence of antibodies was: 24.3% to *B. canis *(n = 1,138), 12.2% to *L. infantum *(n = 569) and 10.1% to *E. canis *(n = 492). Microfilariae and *H. canis *were detected in 372 (7.7%) and 133 dogs (2.2%), respectively. Antibodies to *A. phagocytophilum *were detected in 17.8% (n = 334) out of 1862 tested dogs, *B. burgdorferi *in 30.2% (n = 64) of 212 dogs and *R. conorii *in 34.5% (n = 20) of 58 dogs. The results are illustrated in Figure 1.

### Results of the 331 examined dog samples from Portugal

**Figure 2:**
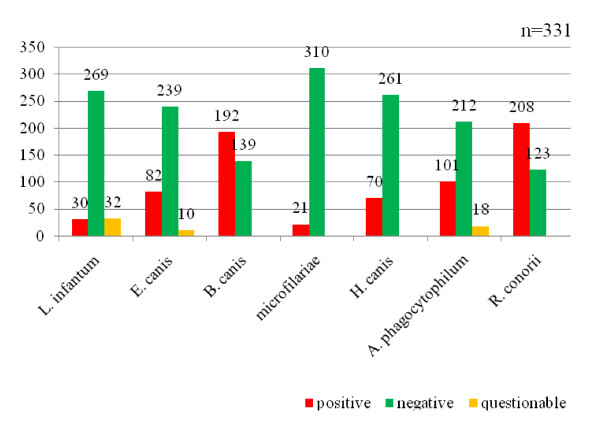
**Number of pathogens detected by IFAT, PCR and Knott's test in 331 autochthonous dogs from kennels/shelters in Portugal**. Number of positive, negative and questionable test results of a total of 331 dogs from Portugal. Blood samples were examined by means of Knott's test for microfilariae and on *H. canis *with the help of the Polymerase chain reaction (PCR). The seroprevalences of *A. phagocytophilum, B. canis, E. canis, L. infantum *and *R. conorii *were determined by means of Immunofluorescence Antibody Test (IFAT).

From the total of 331 autochthonous Portuguese dogs tested, 208 showed antibodies to *R. conorii *(68.2%). The prevalence of the other antibodies detected was: 58% to *B. canis *(n = 192), 30.5% to *A. phagocytophilum *(n = 101), 24.8% to *E. canis *(n = 82) and 9.1% to *L. infantum *(n = 30). Using PCR to detect DNA for *H. canis*, 70 dogs had a positive result (21.1%). Screening the buffy coats, we detected gamonts of *H. canis *in 62 of the samples (18.7%). With the help of the Knott's test we found microfilariae in 21 samples (5.3%). The results are summarized in Figure 2. With help of the acid phosphatase staining and morphological surveys, 8 microfilariae of the species *Acanthocheilonema *(*Dipetalonema*)*dracunculoides*, 7 of *Dirofilaria immitis *and 6 of *Acanthocheilonema *(*Dipetalonema*)*reconditumm *were detected in the dog samples.

### Single and multiple infections in German and Portuguese dogs

**Figure 3:**
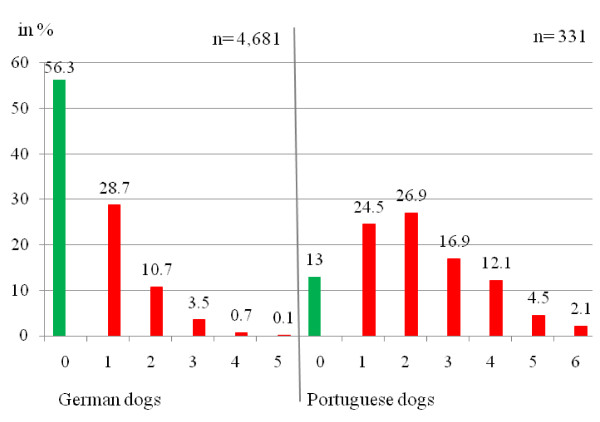
**Single and multiple infections detected by IFAT, PCR, BC and Knott's test in 4,681 German and 331 Portuguese dogs**. Percentage of single, double and multiple infections left from altogether 4,681 German dogs and right from 331 Portuguese dogs.

In both the German and Portuguese dogs double and even multiple CVBD infections were detected. In 56.3% of the German dogs investigated (n = 2,637) no antibodies or pathogens were found. In 28.7% of the dogs, antibodies or one pathogen could be detected (n = 1,341). Altogether in 10.7% an infection with two pathogens (n = 502) was found. In 4.3% of the dogs an infection with more than two pathogens (n = 201) was determined. In contrast to the data from the German dogs, 26.9% of the Portuguese dogs had an infection with two pathogens (n = 89) and in 35.6% of the dogs (n = 118) multiple infections could be detected. Only in 43 dogs (13%) no antibodies or pathogens could be detected. These data are shown in Figure 3.

## Discussion

The study reported here was conducted to evaluate the health status of dogs living in Germany that had either traveled to or were imported from CVBD endemic regions and a comparison was made with an autochthonous Portuguese group of dogs. The results of the 4,681 German dogs clearly indicates that the importation of dogs to Germany is still an explosive topic. Altogether 4,226 dogs were imported to Germany, 2,906 from the Mediterranean area including Portugal. These areas have a considerable prevalence of canine arthropod-borne diseases [5,9,17-24]. Serological testing detects basically chronic and inconspicuous infections and is limited by reduced ability to identify acute infections. In the present study we choose the immunofluorescence antibody test to detect antibodies *to L. infantum, B. canis, E. canis, A. phagocytophilum, R. conorii *and *B. burgdorferi*. Many dogs appear to be able to support chronic infection with vector-borne pathogens for months or even years without displaying obvious deleterious effects [25]. In most cases, dogs without clinical signs and without acute infections, are imported to Germany mostly by animal welfare organizations. With the IFAT, we were aiming to detect clinically inconspicuous infections, in dogs that can be infected with one or even more pathogens. These asymptomatic carriers play a very important role in the epidemiology of zoonotic infection as they are still infectious to the vectors.

*B. canis *was with 1,158 dogs (24.3%) the most diagnosed for German dogs followed by *L. infantum *(12.2%), *E. canis *(10.1%) and infections with microfilariae (7.7%) and *H. canis *(2.2%). In contrast *R. conorii *is the most detected antigen in the Portuguese dogs (68.2%) followed by *B. canis *(58%), *A. phagocytophilum *(30.5%), *E. canis *(24.8%), *H. canis *(21.1%), *L. infantum *(9.1%) and microfilariae (5.3%). Differences between the German and Portuguese dogs can caused by the wide spectrum of countries of origin and destinations dogs travelled to. The spectrum of pathogens and vectors differs in different countries. For example *Hepatozoon *is detected just in 0,7% of 153 examined dogs from Greece [9] but in 48% of 301 examined foxes in Portugal [23]. These data are similar to the number of *H. canis *detected in the 331 Portuguese dogs. *Rickettsia *and *Anaplasma *data are only available for 58 and 1862 German dogs. They could be more similar to the Portuguese results if more samples were detected.

DNA of *H. canis *was examined in 70/331 dogs from Portugal but only in 62 of the examined 331 buffy coat smears gamonts of *H. canis *could be detected. Infections with a low rate of gametocyte-containing leucocytes are difficult to detect, that could be a reason why in 28 samples *H. canis *DNA is found via PCR but no gamont in the buffy coats. But there are 20 cases with definitive diagnosis of *H. canis *gamonts in the blood smears and no findings of DNA via PCR. So it is advisable to employ various diagnostic techniques to achieve a definitive etiological diagnosis of CVBDs, whenever available and economically feasible [26].

Altogether, in 10.2% of the German dogs and in 26.9% of the Portuguese dogs, an infection with two pathogens could be detected. In 4.3% of the dogs from Germany and in 35.6% of the dogs from Portugal multiple infections were found. This indicates that multiple infections are frequent within imported pets - and probably also within pets taken abroad. Clinical signs of dogs infected with more than one pathogen are often non-specific and very variable, such as wasting, weight loss, fever and poor appetite or anorexia, making a definite diagnosis difficult [27].

All in all, dog-tourism and -import confront practicing veterinarians increasingly with rare or still unknown arthropod-borne diseases. In addition, the expanding import and the travelling of dogs can lead to a spread of pathogens and vectors in Germany. These dogs may act as a source of infection for local and still pathogen-free vector populations. Also there is a risk that imported dogs infested with infected vectors might contribute to the further spread of travel related diseases in Germany [3].

## Conclusions

Frequent investigations - particularly in popular holiday destinations - are important to estimate the local risk. For the corresponding countries, specific methods in prophylaxis, diagnostics and therapy must be elaborated.

The consultation of pet-owners with a veterinarian prior to importation of a dog or a journey with their pets to endemic regions is important to either limit importation or establish preventative measures prior to traveling. Prophylactic measures must be in place against vectors, to reduce the likelihood of transmission of vector-borne pathogens, like ectoparasiticides with repellent properties. It would be advisable to create a European recording system for translocation of dogs that register every departure and entry of pets. Standardized serology and ecto- and endoparasiticidal treatments before a re-entry to a non-endemic area should be regularized, like in the United Kingdom [1].

## Methods

During the period of July 2004 to December 2009 blood samples of 4,681 dogs were sent in mostly for random examinations by welfare organizations and private persons via veterinary practitioners. The samples were not accompanied by a case history of the dogs, nor is any information available on the health status. The dog samples examined serological for the following pathogens: *L. infantum, B. canis *and *E. canis*. All samples were examined for microfilariae using the Knott's test and buffy coats were detected for gamonts of *H. canis*. 1,862 of the sample were examined serological additional for *A. phagocytophilum*, 212 samples for *B. burgdorferi *and 58 samples for *R. conorii*.

In the autumn of 2007 and 2008, altogether blood samples of 331 dogs from kennels and shelters from the western part of Algarve/Portugal were collected. Blood samples were collected from brachial veins, 1 ml kept for the Knott's test and centrifuged at 1000 × g for 5 min. Buffy coat smears were exposed, sera separated and stored at -20°C. The dog samples examined serological for the following pathogens: *L. infantum, B. canis, E. canis, A. phagocytophilum *and *R. conorii*. The samples were examined for microfilariae using the Knott's test and for *H. canis *via PCR and screening the buffy coats.

All examinations were conducted in the same laboratory with the same methods, except the *H. canis *PCR.

### Direct pathogen evidence - Knott's test, Buffy Coat, PCR

All EDTA samples were screened for the presence of microfilariae using a modified Knott's test [28]. For the modified Knott's test, 1 ml EDTA blood is mixed with 5 ml of 2% formaldehyde solution in a 15 ml centrifuge tube and centrifuged at 400 × g for 5 min. The supernatant is discarded. The sediment is transferred to glass slides, covered with coverslips and examined by light microscopy at ×10 and ×40 magnifications. Positive Knott's tests were evaluated with the help of the acid phosphatase staining (1.16304.0002. LEUCOGNOST^® ^SP, Merck, Darmstadt, Germany) following the manufacturer's instructions.

For creation of the buffy coats, the blood was centrifuged (1000 × g for 5 min), buffy coat was removed and exposed on glass slides. Buffy coats were stained with May Grünwald's Giemsa (Merck, Darmstadt, Germany) and examined by light microscopy at ×40 magnification.

Samples of the 331 Portuguese dogs were examined additionally via a Polymerase Chain Reaction (PCR) on *H. canis *at the laboratory Laboklin GmbH & Co. KG (Bad Kissingen, Germany) according to their established method.

### Indirect pathogen evidence - IFAT

Immunofluorescence Antibody Test (IFAT) was performed by using commercial kits for *L. infantum*, *B. canis, E. canis, A. phagocytophilum, R. conorii *and *B. burgdorferi *(MegaScreen FLUOLEISH^®^, d4170-L, MegaScreen FLUOBABESIA canis^®^, 19017-Q, MegaScreen FLUOEHRLICHIA canis^®^, d0640-S, MegaScreen FLUOANAPLASMA ph.^®^, 11211-N, MegaScreen FLOURICKETTSIA con.^® ^10447-I, MegaScreen FLUOBORRELIA dog^®^, d1560-L, - Mega Cor Diagnostik GmbH, Hörbranz, Austria). The slides were exposed to sera diluted (1:50) in phosphate buffer solution (PBS, pH 7.2) in a moist chamber and, after washing, to fluorescence labeled anti-dog IgG conjugate (anti-dog IgG, MegaCor, Diagnostik GmbH, Hörbranz, Austria); both incubations were at 37°C for 30 min. Slides were observed under a fluorescence microscope at ×40 magnifications and samples were scored positive when they produced cytoplasmatic inclusion bodies fluorescence. The positive cut-off adopted was at a dilution of 1:50 and all positive sera were titred.

## Competing interests

The authors declare that they have no competing interests.

## Authors' contributions

All the authors have contributed substantially to this study. BM, SL and TJN designed the field studies and carried out the laboratory studies. BM and SL participated in the field studies. BM drafted the manuscript. All authors read and approved the final manuscript.

## References

[B1] Shaw SE Day MJ Birtles RJ Breitschwerdt EB Tick-borne infectious diseases of dogs Trends Parasitol 2001 17 74 80 10.1016/S1471-4922(00)01856-0 11228013

[B2] Otranto D Dantas-Torres F Breitschwerdt EB Managing canine vector-borne diseases of zoonotic concern: part one Trends Parasitol 2009 25 157 163 10.1016/j.pt.2009.01.003 19269898

[B3] Glaser B Gothe R Imported arthropod-borne parasites and parasitic arthropods in dogs: Spectrum of species and epidemiological analysis of the cases diagnosed in 1995 and 1996 Tierärztl Prax 1998 26 40 46 9531673

[B4] Glaser B Gothe R Tourism and import of dogs: An inquiry in Germany on the extent as well as on the spectrum and preference of countries concerning stay abroad and origin, respectively Tierärztl Prax 1998 26 197 202 9646416

[B5] Weise M Relevant species of the canine parasite fauna in European Mediterranean countries and Portugal for dogs in Germany concerning epidemiology and travel veterinary medicine - a literature review PhD thesis 2004 University Munich, Faculty of Veterinary Medicine

[B6] Beelitz P Pfister K Diagnosis and treatment of exotic diseases in travelling dogs Tierärztl Prax 2004 32 158 165

[B7] Hirsch M Pantchev N Occurrence of the travel diseases leishmaniosis, ehrlichiosis, babesiosis and dirofilariosis in dogs living in Germany Kleintierprax 2008 53 154 165

[B8] Defra department for Environment Food and Rural Affairs http://www.defra.gov.uk/wildlife-pets/pets/travel/pets/procedures/stats.htm

[B9] Jensen J Müller E Daugschies A Arthropod-borne diseases in Greece and their relevance for pet tourism Prakt Tierarzt 2003 84 430 438

[B10] Daugschies A Import of parasites by tourism and animal trading Dtsch Tierärztl Wschr 2001 108 348 352 11560118

[B11] Deplazes P Staebler S Gottstein B Travel medicine of parasitic diseases in the dog Schweiz Arch Tierheilk 2006 9 447 461 10.1024/0036-7281.148.9.447 17024974

[B12] Gärtner S Just FT Pankraz A *Hepatozoon canis *infections in two dogs from Germany Kleintierprax 2008 53 81 87

[B13] Naucke TJ Menn B Massberg D Lozentz S Sandflies and leishmaniasis in Germany Parasitol Res 2008 103 Suppl 1 65 68 10.1007/s00436-008-1052-y 19030887

[B14] Jensen J Simon D Schaarschmidt-Kiener D Müller W Nolte I Retrospective assessment of autochthone infection with *Ehrlichia canis *in dogs in Germany Tierärztl Prax 2007 35 123 128

[B15] Hermosilla C Pantchev N Dyachenko V Gutmann M Bauer C First autochthonous case of canine ocular *Dirofilaria repens *infection in Germany Vet Rec 2006 158 134 135 16443841 10.1136/vr.158.4.134

[B16] Pantchev N Norden N Lorentzen L Rossi M Rossi U Brand B Dyachenko V Current Surveys on the Prevalence and Distribution of *Dirofilaria *spp. in Dogs in Germany Parasitol Res 2009 105 63 74 10.1007/s00436-009-1497-7 19575227

[B17] Solano-Gallego L Lull J Osso M Hegarty B Breitschwerdt E A serological study of exposure to arthropod-borne pathogens in dogs from northeastern Spain Vet Res 2006 37 231 244 10.1051/vetres:2005054 16472522

[B18] Tabar MD Francino O Altet L Sánchez A Ferrer L Roura X PCR survey of vectorborne pathogens in dogs living in and around Barcelona, an area endemic for leishmaniosis Vet Rec 2009 164 112 116 19168881 10.1136/vr.164.4.112

[B19] Torina A Caracappa S Dog tick-borne diseases in Sicily Parassitologia 2006 48 145 147 16881419

[B20] Otranto D Dantes-Torres F Canine and feline vector-borne diseases in Italy: current situation and perspectives Parasit Vectors 2010 3 2 10.1186/1756-3305-3-2 20145730 PMC2818618

[B21] Cardoso L Costa A Tuna J Vieira L Eyal O Yisaschar-Mekuzas Y Baneth G *Babesia canis canis *and *Babesia canis vogeli *infections in dogs from northern Portugal Vet Parasitol 2008 156 199 204 10.1016/j.vetpar.2008.05.027 18602757

[B22] Alexandre N Santos AS Núncio MS Sousa R Boinas F Bacellar F Detection of *Ehrlichia canis *by polymerase chain reaction in dogs from Portugal Vet J 2009 181 343 344 10.1016/j.tvjl.2008.03.025 18682335

[B23] Conceição-Silva FM Abranches P Silva-Pereira MCD Janz G Hepatozoonosis in foxes from Portugal J Wildl Dis 1988 24 344 347 3373641 10.7589/0090-3558-24.2.344

[B24] Cortes S Afonso MO Alves-Pires C Campino L Stray Dogs and Leishmaniasis in Urban Areas, Portugal Emerg Infect Dis 2007 13 1431 1432 18252134 10.3201/eid1309.070101 PMC2857284

[B25] Breitschwerdt EB Canine and feline anaplasmosis: Emerging infectious diseases Proceedings of the 2nd Canine Vector-Borne Disease (CVBD) Symposium: 25-28 April 2007; Sicily, Italy 2007 6 14

[B26] Otranto D Dantas-Torres F Breitschwerdt EB Managing canine vector-borne diseases of zoonotic concern: part two Trends Parasitol 2009 25 228 235 10.1016/j.pt.2009.02.005 19346164

[B27] Kordick SK Breitschwerdt EB Hegarty BC Southwick KL Colitz CM Hancock SI Bradley JM Rumbough R Mcpherson JT MacCormack JN Coinfection with multiple tick-borne pathogens in a Walker Hound kennel in North Carolina J Clin Microbiol 1999 37 2631 2638 10405413 10.1128/jcm.37.8.2631-2638.1999 PMC85300

[B28] Euzeby J Diagnostic Expérimental des Helminthoses animales livre 1 Boulevard de Grenelle. Paris 1981 277 312

